# Supplementary material for: Model-based fed-batch cultivation of Viola odorata plant cells exhibiting antimalarial and anticancer activity
Source: Front Bioeng Biotechnol. 2025 Jan 29;13:1528570. doi: 10.3389/fbioe.2025.1528570 (PMC11815595; doi:10.3389/fbioe.2025.1528570)
Supplement: Supplementary file 1 [file DataSheet1.docx]

Supplementary Material

# Supplementary Figures and Tables

## Supplementary Figures


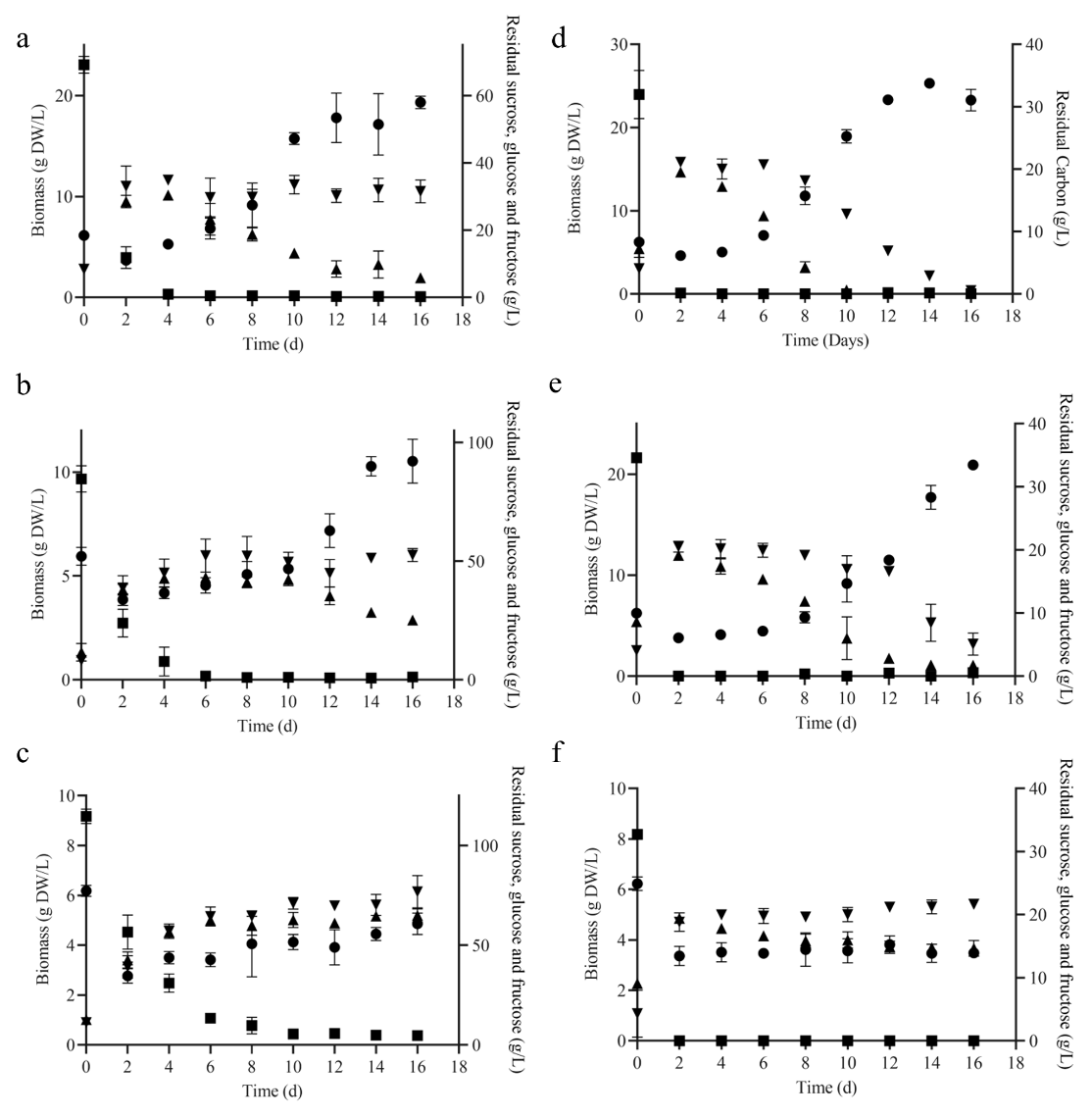


**Supplementary Figure S1**. Batch growth kinetics of *V. odorata* cells on optimized medium composition supplemented with sucrose concentration 75 g L^-1^ (a), 105 g L^-1^ (b), 145 g L^-1^ (c) and with potassium nitrate concentration 4 g L^-1^ (d), 8 g L^-1^ (e) and 12 g L^-1^ (f). Concentration profiles of biomass (g DW L^-1^*,* ●), sucrose (g L^-1^*,* ■), glucose (g L^-1^*, ▲*), and fructose (g L^-1^*, ▼*),


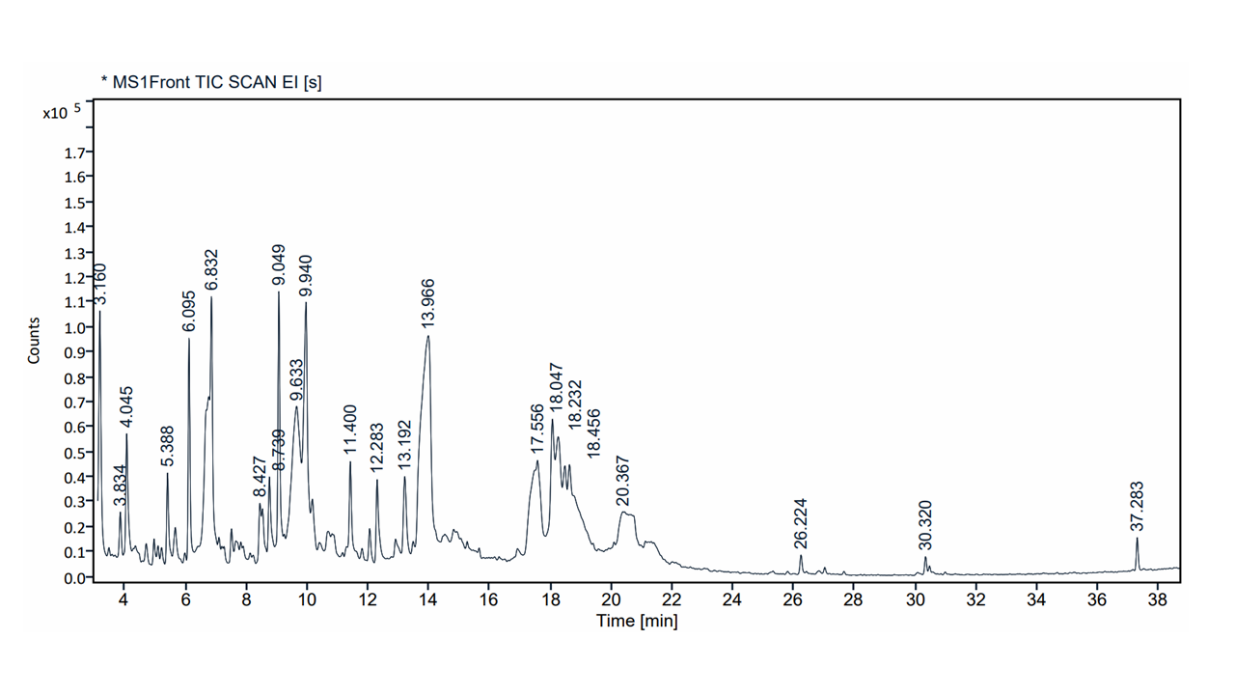


**Supplementary Figure S2**. Full scan GCMS chromatograms of *V. odorata* biomass extracts obtained from STR operated in fed batch mode of cultivation

## Supplementary Table

**Supplementary Table S1:** Primary and secondary metabolites identified from *V. odorata* biomass extracts obtained from STR operated in fed batch mode of cultivation using GCMS

| **S. No** | **RT** | **Compound name** |
| --- | --- | --- |
|  | 3.160 | 2-Cyclopenten-1-one, 2- hydroxy- |
|  | 3.834 | 2,4-Dihydroxy-2,5- dimethyl-3(2H)-furan-3- one |
|  | 4.045 | 2(5H)-Furanone, 5-ethyl- |
|  | 5.388 | 1,3,4,6,7,8-Hexahydro-1- methyl-2H-pyrimido[1,2- a]pyrimidine |
|  | 6.095 | cis-Aconitic acid |
|  | 6.832 | 4H-Pyran-4-one, 2,3- dihydro-3,5-dihydroxy-6- methyl- |
|  | 8.427 | 1,2,6-Hexanetriol |
|  | 8.739 | 5-Hydroxymethylfurfural |
|  | 9.049 | L-Sorbose |
|  | 9.633 | D-Galactose |
|  | 9.940 | 5-(2-Hydroxyethyl)-4- methylthiazole |
|  | 11.400 | Isosorbide Dinitrate |
|  | 12.283 | 1H-Pyrazole-3-acetic acid, 2,5-dihydro-5-oxo |
|  | 13.192 | Butanamide, 2-hydroxy-N,2,3,3-tetramethyl- |
|  | 13.966 | Sucrose |
|  | 17.556 | 3-Deoxy-d-mannoic lactone |
|  | 18.047 | Trehalose |
|  | 18.232 | d-Glycero-d-ido-heptose |
|  | 18.456 | d-Mannose |
|  | 20.367 | β-D-Glucopyranose |
|  | 26.224 | n-Hexadecanoic acid |
|  | 30.320 | 1,E-11,Z-13- Octadecatriene |
|  | 37.283 | Hexadecanoic acid, 2- hydroxy-1- |
